# Supplementary material for: Revisiting Public Trust and Media Influence During COVID-19 Post-Vaccination Era—Waning of Anxiety and Depression Levels Among Skilled Workers and Students in Serbia
Source: Behav Sci (Basel). 2025 Jul 11;15(7):939. doi: 10.3390/bs15070939 (PMC12292197; doi:10.3390/bs15070939)
Supplement: Supplementary file 1 [file behavsci-15-00939-s001.zip › behavsci-3655280-supplementary.pdf]

Public Trust and Media Influence During COVID-19

Questionnaire

I

Gender

1. Female
2. Male

Age (years)

____

Highest levels of education

1. High school
2. Bachelor degree
3. Master of Science
4. Doctorate level

Population size of the residence location:

1. Less than 10,000
2. 10,000-50,000
3. 50,000-100,000
4. 100,000-500,000
5. More than 500,000

Occupation:

1. Healthcare – physician
2. Healthcare – nurse/medical technician
3. Education – elementary school teacher
4. Education – high school teacher/professor
5. Education – university professor
6. Military personnel

II

Below is a list of common symptoms of anxiety. Please carefully read each item in the list. Indicate how much you have been bothered by that symptom during the past month, including today, by circling the number in the corresponding space in the column next to each symptom.

Item	Symptom	Not at all	Mildly	Moderately	Severely
1	Numbness or tingling				
2	Feeling hot				
3	Wobbliness in legs				
4	Unable to relax				
5	Fear of the worst happening				
6	Dizzy or lightheaded				
7	Heart pounding/racing				
8	Unsteady				
9	Terrified or afraid				
10	Nervous				
11	Feeling of choking				
12	Hands trembling				
13	Shaky/unsteady				
14	Fear of losing control				
15	Difficulty breathing				
16	Fear of dying				
17	Scared				
18	Indigestion or abdominal discomfort				
19	Faint/lightheaded				
20	Face flushed				
21	Hot/cold sweats				

To which degree has COVID-19 pandemic influenced the responses you provided to the previous question?

1. Smallest degree of influence
2. Very small degree of influence
3. Somewhat small degree of influence
4. Somewhat high degree of influence
5. Very high degree of influence
6. Highest degree of influence

III

For each item below, please check the column which best describes how often you felt or behaved this way during the past several days.

Item	Statement	A little of the time	Some of the time	Good part of the time	Most of the time
1	I feel down-hearted and blue.				
2	Morning is when I feel best.				
3	I have crying spells or feel like crying.				
4	I have trouble sleeping at night.				
5	I eat as much as I used to.				
6	I still enjoy sex.				
7	I notice I am losing weight.				
8	I have trouble with constipation.				
9	My heart beats faster than usual.				
10	I get tired for no reason.				
11	My mind is as clear as it used to be.				
12	I find it easy to do things I used to.				
13	I am restless and can’t keep still.				
14	I feel hopeful about the future.				
15	I am more irritable than usual.				
16	I find it easy to make decisions.				
17	I feel I am useful and needed.				
18	My life is pretty full.				
19	I feel others would be better off if I died.				
20	I still enjoy things I used to do.				

To which degree has COVID-19 pandemic influenced the responses you provided to the previous question?

1. Smallest degree of influence
2. Very small degree of influence
3. Somewhat small degree of influence
4. Somewhat high degree of influence
5. Very high degree of influence
6. Highest degree of influence

IV

**During the outbreak, have you experienced disturbance by**	Never	Very rarely	Rarely	Sometimes	Usually	Often
media reports regarding the outbreak?						
the information from other sources you have learned on your own initiative?						
the lack of the information regarding the COVID-19 outbreak and the disease itself?						
the possibility of virus transmission from other people despite personal preventive measures you applied?						

**Compared to the period before the implementation of vaccination, how do you describe the level of psychological disturbance by**	It decreased	It did not change	It increased
media reports regarding the outbreak?			
the information from other sources you have learned on your own initiative?			
the lack of the information regarding the COVID-19 outbreak and the disease itself?			
the possibility of virus transmission from other people despite personal preventive measures you applied?			

V

**During the outbreak, have you expressed trust in**	Never	Very rarely	Rarely	Sometimes	Usually	Often
the healthcare system?						
the preventive measures proposed by the Crisis team?						

**Compared to the period before the implementation of vaccination, how do you describe the level of trust you expressed in**	It decreased	It did not change	It increased
the healthcare system?			
the preventive measures proposed by the Crisis team?			
